# Detailed analysis of the association between urate deposition and bone erosion in gout: a dual-energy computed tomography study

**DOI:** 10.3389/fendo.2023.1167756

**Published:** 2023-04-17

**Authors:** Wan-Yi Zheng, Wen-Feng Zhan, Jing-Yi Wang, Wei-Ming Deng, Yu-Kai Hung, Wei Wang, Gui-Hua Jiang

**Affiliations:** ^1^ Department of Medical Imaging, The Affiliated Guangdong Second Provincial General Hospital of Jinan University, Guangzhou, China; ^2^ Department of Medical Imaging, Guangdong Second Provincial General Hospital, Guangzhou, China; ^3^ United Imaging Healthcare, Shanghai, China; ^4^ Department of Rheumatology and Immunology, Guangdong Second Provincial General Hospital, Guangzhou, China

**Keywords:** gout, dual-energy computed tomography, urate deposition, bone erosion, serum uric acid

## Abstract

**Objective:**

This study aimed to analyze the effect of urate deposition (UD) on bone erosion and examine the association between the volume of monosodium urate (MSU) crystals and an improved bone erosion score method, as measured in the metatarsophalangeal (MTP) joints of patients with gout.

**Materials and methods:**

Fifty-six patients diagnosed with gout using the 2015 European League Against Rheumatism and American College of Rheumatology criteria were enrolled. MSU crystals volume at each MTP joint was measured using dual-energy computed tomography (DECT) images. The degree of bone erosion was evaluated with the modified Sharp/van der Heijde (SvdH) erosion scoring system based on CT images. Differences in clinical features between patients with (UD group) and without (non-UD group) UD were assessed, and the correlation between erosion scores and urate crystal volume was analyzed.

**Results:**

The UD and non-UD groups comprised 30 and 26 patients, respectively. Among the 560 MTP joints assessed, 80 showed MSU crystal deposition, and 108 showed bone erosion. Bone erosion occurred in both groups but was significantly less severe in the non-UD group (*p <*0.001). Both groups had equivalent levels of serum uric acid (*p*=0.200). Symptom duration was significantly longer in the UD group (*p*=0.009). The UD group also had a higher rate of kidney stones (*p*=0.023). The volume of MSU crystals was strongly and positively associated with the degree of bone erosion (r=0.714, *p <*0.001).

**Conclusion:**

This study found that patients with UD show significant increased bone erosion than those without UD. The volume of MSU crystals is associated with the improved SvdH erosion score based on CT images, regardless of serum uric acid level, demonstrating the potential of combining DECT and serum uric acid measurements in helping optimize the management of patients with gout.

## 1 Introduction

Gout is a disease caused by the deposition of monosodium urate (MSU) crystals, which can lead to erosive destructive arthropathies and even disability (1–3). Tophus is considered the strongest risk factor for joint damage (4), and the gold standard for gout diagnosis is the microscopic identification of MSU crystals in tophi or joint synovial fluid aspirations (5). The deposition of MSU crystals results from high serum urate concentration and exceeds the physiological saturation point. Serum uric acid level is an indicator for the diagnosis of gout and evaluation of the efficacy of urate-lowering therapy (ULT) (5, 6). However, a one-time measurement of serum uric acid levels cannot accurately reflect disease severity or changes in bone structure with ULT. Furthermore, the current methods for the identification and evaluation of gout are invasive. Serum uric acid is a dynamic metabolite that can be affected by ULT (7). Therefore, a non-invasive, stable, and reproducible method is needed to detect and evaluate MSU crystals and bone erosion.

In clinical practice, radiology is employed to non-invasively detect bone erosion and MSU crystals in patients with gout (8, 9). The currently used Sharp/van der Heijde (SvdH) scoring method for bone erosion is based on plain radiography and was proposed in 1971 (10). However, plain radiography has limitations, as it does not show erosions or tophi until the disease has progressed to a relatively late stage, and variations in joint positioning can affect the assessment (11, 12). Computed tomography (CT) provides three-dimensional information for more accurate assessments of erosions in the foot without the shortcoming of plain radiography, but it cannot characterize crystal depositions (13). Dual-energy computed tomography (DECT) is a developed CT method that allows for the detection of bone erosion, as well as the specific detection and volume measurement of urate crystals (14). Therefore, the classification criteria recommended by the 2015 European League Against Rheumatism (EULAR) and the American College of Rheumatology (ACR) endorsed DECT as a novel and effective diagnostic tool for gout (5).

Bone erosion is a strong predictor of musculoskeletal disability in gout (15); therefore, early detection and assessment are critical in reducing the risk of joint functional disability. Over the past decade, radiology has been used to investigate the relationship between MSU crystals and bone erosion (16–19). Jin et al. (18) employed a novel semi-quantitative DECT scoring system to assess urate deposition (UD) and demonstrate that total urate was associated with bone erosion. Mark et al. (16) utilized a plain radiograph-erosion score and urate volume from DECT to demonstrate that tophus urate was directly associated with erosion. However, their methods were rather not highly sensitive and precise. To the best of our knowledge, no study has assessed erosion using the SvdH scores based on CT images. Therefore, investigating the relationship between the volume of MSU crystals and degree of bone erosion (bone destruction) based on CT imaging could be of great value in the prevention and early detection of gout progression and joint disability.

The aim of this study is to analyze the influence of tophus in the development of bone destruction and to investigate the relationship between the volume of MSU crystals and bone erosion using the improved SvdH score based on CT images.

## 2 Materials and methods

### 2.1 Study population

This study was approved by the Institutional Review Board of our hospital (approval number: 2022-KY-KZ-227-02). The need for informed consent was waived due to the retrospective nature of the study.

The inclusion criteria were patients who 1) had a history or presence of clinical manifestation of podagra; 2) underwent clinical evaluation for gout and had a 2015 EULAR/ACR classification criteria score >8 (5); 3) received ULT; and 4) underwent DECT scanning of both feet. Sixty-two patients met these criteria between September 2021 and November 2022. Each joint of the patient was considered as an individual sample to explore the relationship between the volume of MSU crystals and erosion score.

**Figure 1:**
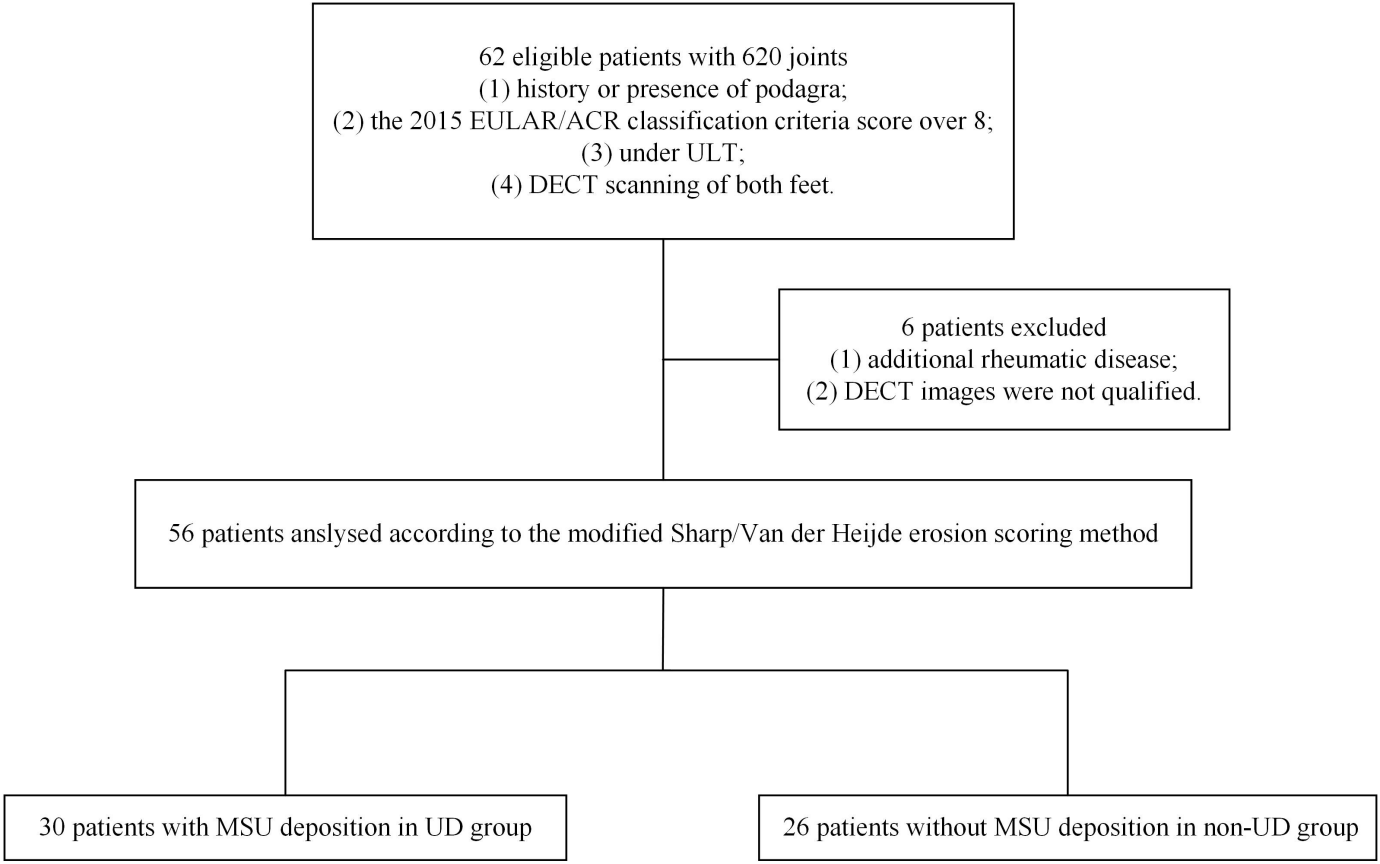
Flowchart illustrating the selection of patients and joints. DECT, dual-energy computed tomography; EULAR/ACR, European League Against Rheumatism/American College of Rheumatology; MSU, monosodium urate; UD, urate deposition; ULT, urate-lowering therapy.

The exclusion criteria were 1) co-existing rheumatic disease or lower limb amputations and 2) unqualified DECT images. Six patients were excluded from the study (**Figure 1**). Final diagnoses of gout were made by two rheumatologists in consensus (W.D and Y.H) who had 18 and 12 years of experience, respectively, based on the 2015 EULAR/ACR criteria.

### 2.2 Clinical assessment

Demographic data (sex, age, and body mass index [BMI]), clinical data (duration of symptom, alcohol consumption, and smoking), and laboratory findings (serum uric acid levels) were recorded. Normal serum uric acid levels were deemed in the range of 125-410 μmol/L. BMI was calculated by dividing weight (kg) by height (m^2^). Clinical information on hypertension, hyperlipidemia, diabetes mellitus (hyperglycemia), renal disease (history of kidney stone), and chronic heart disease was also collected. A DECT scan was performed within 3 days of the collection of clinical data.

### 2.3 DECT acquisition and reconstruction

All CT scans were performed using a single source DECT system in axial mode (uCT 960+, United Imaging Healthcare) with a tube voltage of 80/140 kV, tube current of 330/80 mAs, rotation time of 0.8 s, and collimation of 160 mm. Patients were scanned in the supine position and craniocaudal direction starting from both ankle joints to the distal big toe, with the foot fixed in plantarflexion.

Axial images of 80 and 140 kV were reconstructed into a 512×512 matrix size and 1-mm slice thickness and were subsequently loaded into a post-processing workstation (uWS-CT: R005 workstation, United Imaging Healthcare) for the automatic identification of MSU crystals with a material decomposition algorithm according to its material-specific dual-energy properties. Mixed images with a composition of 50% (50% 80 kV images mixed with 50% 140 kV images) were generated. A color-coded display was overlaid on the mixed CT and 3D volume-rendering images, where uric acid was marked as green and cortical bone as blue.

### 2.4 Analysis of DECT images

**Figure 2:**
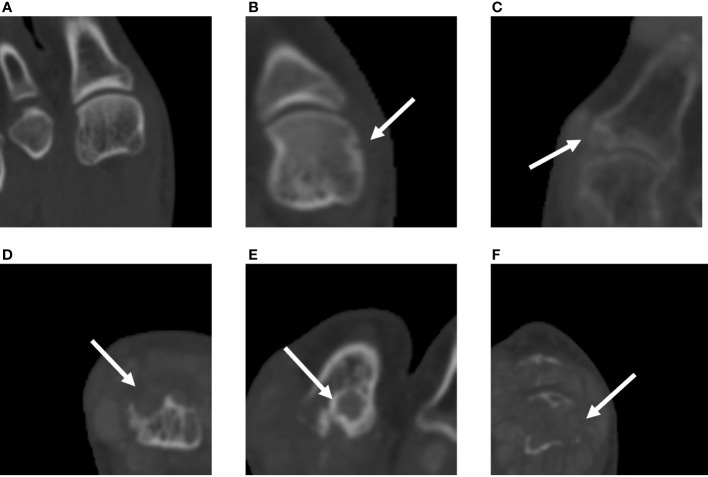
Assessment of bone erosion in the MTP joints with CT images using the modified SvdH erosion scoring method. **(A)** A score of 1 indicates tiny and discrete erosion. **(B)** A score of 2 indicates spread erosion. **(C)** A score of 3 indicates an erosion surface nearly half of the joint surface. **(D)** A score of 4 indicates erosion exceeding half of the joint surface. **(E)** A score of 5 indicates extensive bone erosion and loss. SvdH, Sharp/van der Heijde; CT, computed tomography; MTP, metatarsophalangeal joint.

The study used the SvdH erosion scoring method, which focuses only on the metatarsophalangeal (MTP) joints, to score bone erosion in 10 MTP joints (score 0–10 per joint, 0–5 per articular surface; **Figure 2**). The modified SvdH erosion scoring method scoring is described as follows: score 0, no definite bony erosion; score 1, tiny and discrete erosion; score 2, erosion spread; score 3, erosion surface nearly half of the joint surface; score 4, erosion surface exceeding half of the joint surface; and score 5, extensive erosion. The overall erosion score was defined as the sum of the erosion scores for each joint. The maximum bone erosion score possible was 50 for each foot.

Two radiologists with 9 and 23 years of experience (W.Z. and G.J.) who were blinded to clinical characteristics and laboratory parameters analyzed the bone erosion and the damage score of each joint. Cortical erosion was referred to an area with loss of cortex and sharply defined margins, as observed in two planes, along with a cortical break.

MSU crystals were defined using the following criteria: 1) hyperdense material deposits in the MTP joints on non-contrast CT images and 2) green color-coded voxel deposits in MTP joints on DECT images. Images with common artifacts, including skin artifacts, metal artifacts, beam hardening from dense cortical bone, and motion artifacts, were excluded. UDs were manually outlined by W.Z. at each MTP joint, and the tophi volumes (mm^3^) were automatedly calculated by the post-processing workstation, with an upper limit of 3000 Hounsfield units (HU) and lower limit of 150 HU.

### 2.5 Statistical analyses

All statistical analyses were performed using SPSS version 25 (IBM Corp., Armonk, NY, USA). A *p*-value <0.05 was considered statistically significant, and all results were two-tailed.

Categorical variables were presented as proportions, and continuous variables were summarized using mean ± standard deviation. The Kolmogorov-Smirnov test was used to examine data distribution. The parameters of the two groups were compared using the Mann-Whitney U test for continuous variables and chi-square tests for categorical variables. The intraclass correlation coefficient (ICC) was used to estimate intra-observer agreement for erosion scores, with 0–0.20 representing slight agreement; 0.21–0.40 representing fair agreement; 0.41–0.60 representing moderate agreement; 0.61–0.80 representing substantial agreement; and 0.81–1.00 representing near-perfect agreement. Spearman correlation coefficient and linear regression models were used to determine the correlation between the volume of MSU crystal deposition and bone erosion score.

## 3 Results

### 3.1 Patients’ characteristics and *post hoc* analysis

A total of 56 patients (mean age, 47.2 ± 12.9 years) were finally enrolled in this retrospective study. All patients were male, as gout occurs more frequently in males. To compare the clinical characteristics between the patients with and without UD, they were divided into two groups based on their DECT results: patients who had UD were assigned to the UD group (n=30), and those who did not were assigned to the non-UD group (n=26). Descriptive data on demographics and clinical characteristics are shown in **Table 1**.

**Table 1:** Comparison of patient characteristics in the UD and non-UD groups.

	UD group (N=30)	Non-UD group (N=26)	P value
Age (y)	50.27 ± 12.90	43.69 ± 12.08	0.060
BMI	26.45 ± 2.76	25.80 ± 3.12	0.052
Symptom duration (month)	107.63 ± 69.36	84.64 ± 35.91	0.009*
Overall erosion score	14.27 ± 1.10	1.38 ± 2.25	<0.001*
Serum uric acid/(μmol/L)	498.67 ± 162.26	453.92 ± 130.33	0.200
Alcohol ingestion, N (%)	14 (46.67)	7(26.92)	0.170
Smoke, N (%)	17 (56.67)	8 (30.77)	0.064
Comorbidities			
Hyperlipidemia, N (%)	15 (50.00)	7 (26.92)	0.166
Hypertension, N (%)	8 (26.67)	2 (7.69)	0.087
Hyperglycemia. N (%)	5 (16.67)	1 (3.84)	0.200
History of kidney stone, N (%)	9 (30.00)	0 (0)	0.023*
Chronic heart disease, N (%)	4 (30.77)	0 (0)	0.115

Data is expressed as mean ± standard deviation or count (percentage).

BMI, body mass index; UD, urate deposition.

*Indicates statistical significance.

**Figure 3:**
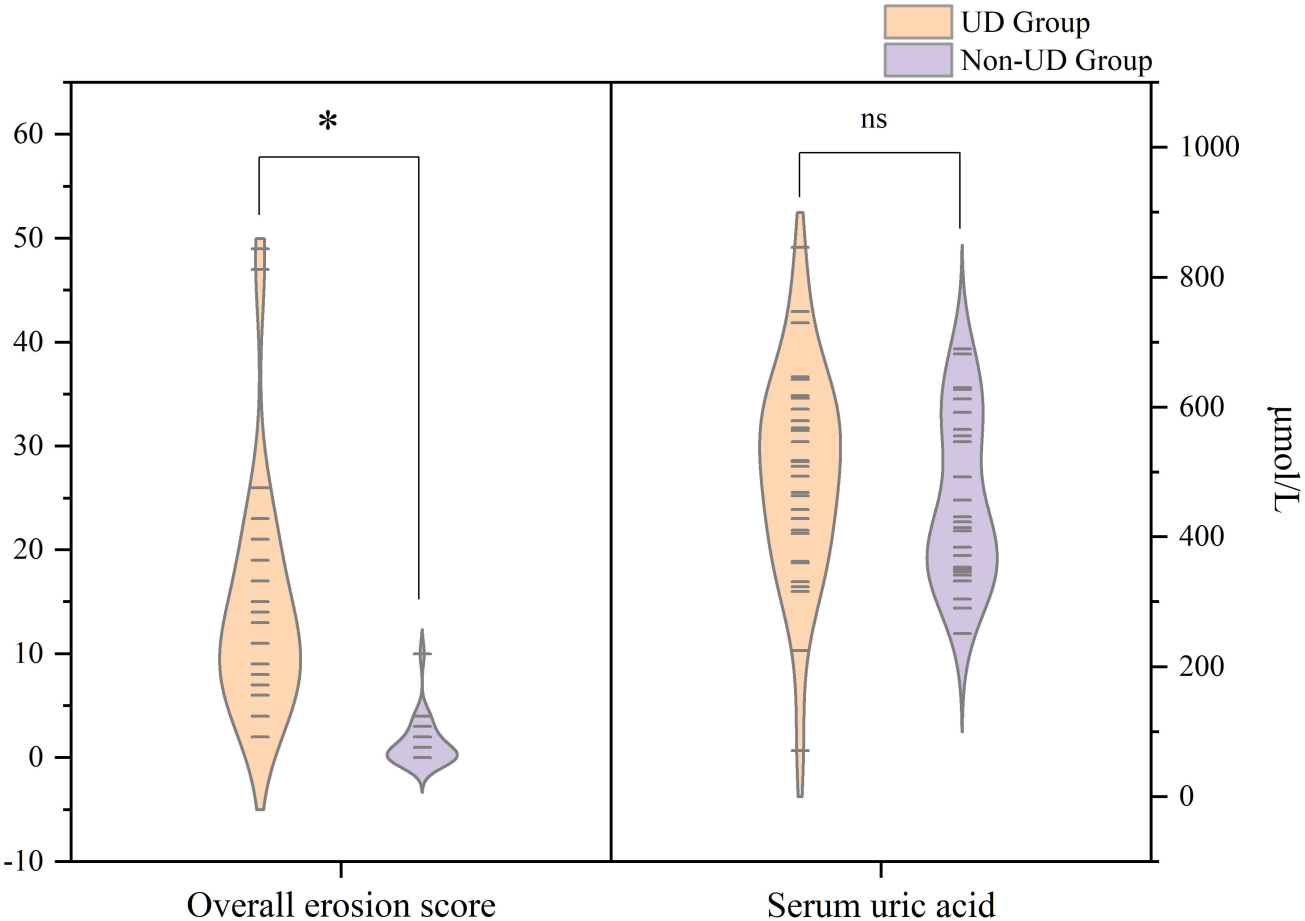
Differences between the UD and non-UD groups in the overall erosion score and serum uric acid. The modified SvdH erosion scores were significantly higher in the UD group than in the non-UD group (left), but serum urate level did not differ between the two groups (right). SvdH, Sharp/van der Heijde; UD, urate deposition.

No statistical difference was found between the UD and non-UD groups in terms of age and BMI (*p*=0.60 and *p*=0.052, respectively). The average serum urate level was 498.67 ± 162.26 μmol/L in the UD group and 453.92 ± 130.33 in the non-UD group (*p*=0.200; **Figure 3**). The prevalence rates of alcohol consumption and smoking were equivalent between both groups (*p*=0.170 and *p*=0.064, respectively). Moreover, there was no difference between the UD and non-UD groups in the prevalence of hyperlipidemia, hypertension, hyperglycemia, and chronic heart disease (all *p >*0.087). In comparison, the duration of the symptoms was significantly longer in the UD group than in the non-UD group (mean=107.63 ± 69.36 and 84.64 ± 35.91 months, respectively; *p*=0.009). The overall erosion score in the UD group was also significantly higher than that in the non-UD group (14.27 ± 1.10 and 1.38 ± 2.25, *p <*0.001; **Figure 3**). Notably, the UD group had a significantly higher rate of kidney stones than the non-UD group (*p*=0.023).

### 3.2 Analysis of tophus burden and bone erosion in joints

**Figure 4:**
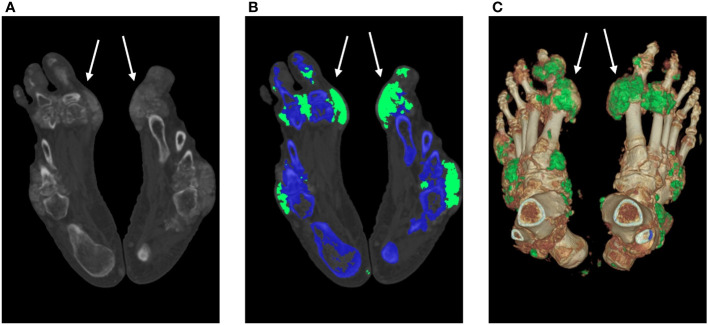
MSU crystal deposition and bone erosion. An MSU crystal was discovered in a 53-year-old man despite modest blood uric acid levels (323 μmol/L). The DECT images illustrate the MSU deposition of the feet (white arrow). A 140-kV CT image showing hyperdense deposition (left); color-coded 50% mixed-energy CT image (middle) showing MSU crystal (green) and bone structure (blue); and surface-rendered three-dimensional CT image (right) showing MSU crystal (green) and bone structure (white). The erosion score is 8 in the left MTP1 joint and 10 in the right MTP1 joint. CT, computed tomography; DECT, dual-energy computed tomography; MSU, monosodium urate; and MTP, metatarsophalangeal joint.

**Figure 5:**
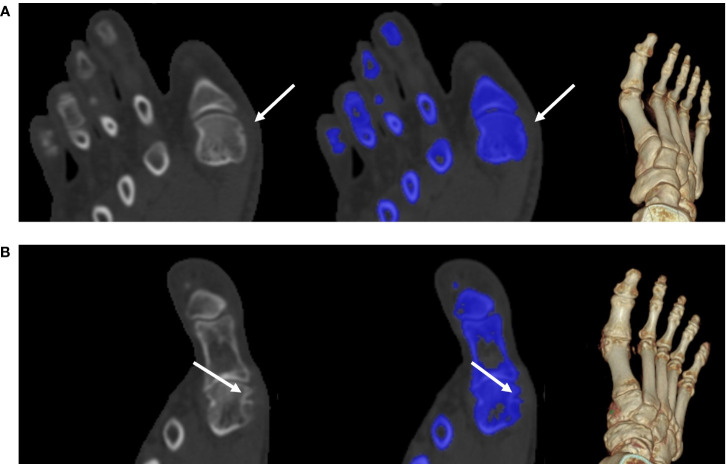
Tiny bone erosion on the medial side of the metatarsal head. **(A)** A 31-year-old man in the non-UD group presented with knee joint ache for two days; his serum urate level was 332 μmol/L. He underwent intermittent medication to control the acute flare, though his response to medication this time was not well. Bone erosion (white arrow) had a score of 1. A 140-kV CT image showing bone erosion (white arrow) (left); color-coded 50% mixed-energy CT image (middle) showing bone erosion (white arrow) and bone structure (blue); and surface-rendered three-dimensional CT image (right). **(B)** A 58-year-old man in the non-UD group presented with chronic gout for approximately 22 years (serum urate, 568 μmol/L). The patient was not adherent to uric acid-lowering medications. Bone erosion (arrow) had a score of 2. A 140-kV CT image showing bone erosion (white arrow) (left); color-coded 50% mixed-energy CT image (middle) showing bone erosion (white arrow) and bone structure (blue); surface-rendered 3D CT image (right). CT, computed tomography; UD, urate deposition.

Bone erosion scores were visually assessed in 560 MTP joints from 56 patients by 2 radiologists, with an overall ICC of 0.87 (95% confidence interval: 0.86–0.97). UDs close to or within the joints were found in 80 MTP joints (14.29%; mean volume=4307.91 ± 6107.62 mm^3^) in 56 subjects. Additionally, 108 MTP joints (19.28%; mean score=4.2 ± 2.5) showed bone erosion. The erosion scores were affected by the distribution of MSU crystal deposition, with the first MTP joint having the highest mean score (mean score=4.6), followed by sites in the second MTP joint (mean score=4). The minimum score was assigned to the fifth MTP joint (mean score=3), and the first MTP joint was the most frequently affected (n=64; 21.33% of MTP joints had UD), whereas the fourth was the least frequently affected (n=3, 1.33% of the MTP joints had UD). A representative case of tophus burden and bone erosion is shown in **Figure 4**. In the non-UD group, erosion was observed in 16 (5.33%) joints of 12 patients. Small bone erosion (score 1 or 2) was more prevalent on the medial side of the metatarsal head (**Figure 5**).

**Figure 6:**
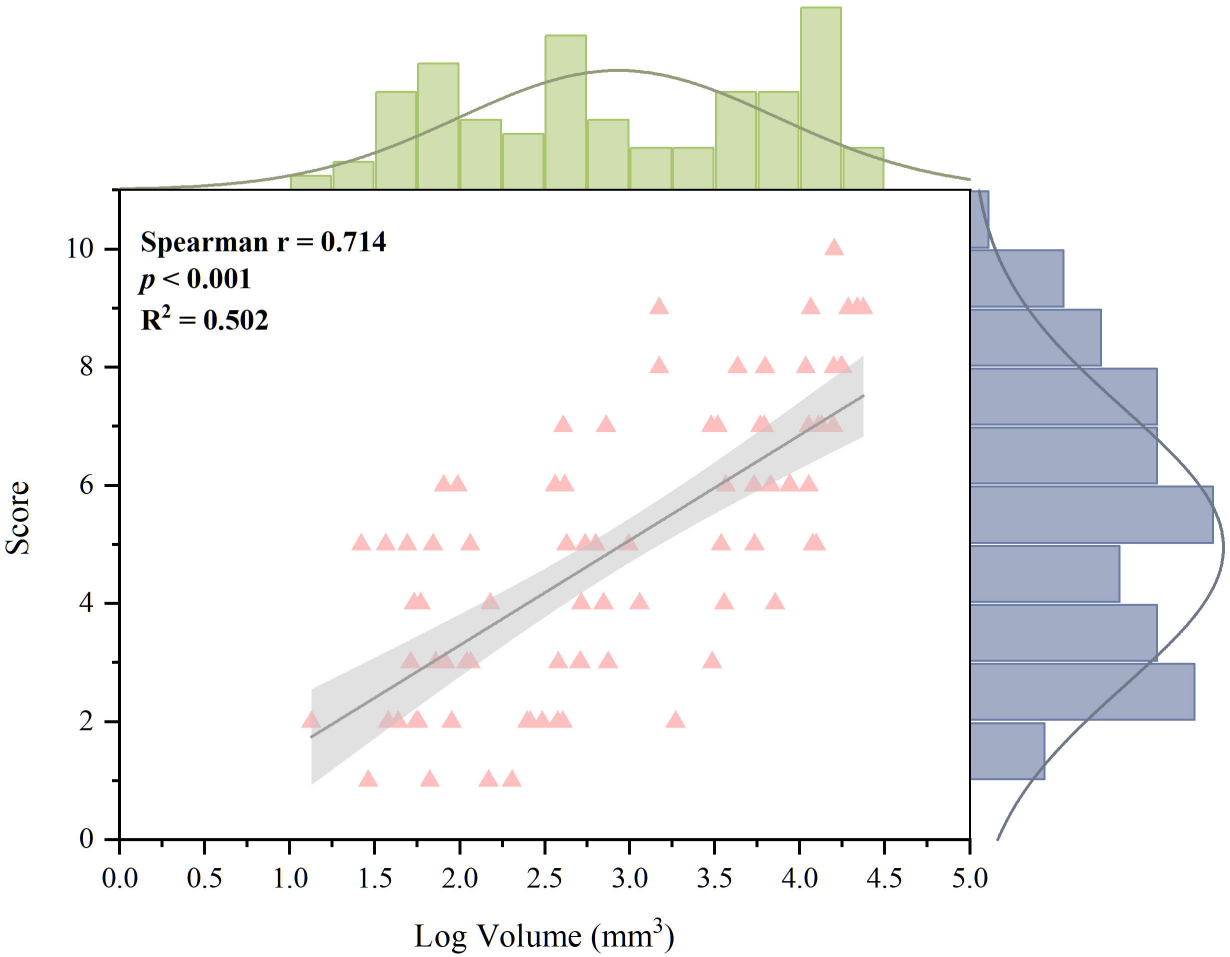
Correlation between erosion score and logged volume of MSU deposition for 80 joints with both tophus burden and bone erosion. A strong and positive relationship was found (Spearman correction coefficient, 0.714; *p <*0.001). MSU, monosodium urate.

The relationship between the volume of MSU crystals and bone erosion score was determined using the 80 MTP joints of 30 patients with both tophus burden and bone erosion. The bone erosion score was positively and strongly associated with the logged tophus volume (r=0.714, p <0.001) (**Figure 6**).

## 4 Discussion

Detection of bone erosion and MSU crystals provides a possibility for the early detection of joint damage, as well as the prevention of and intervention for joint deformity and disability in gout. This study analyzed the CT images of feet and bone destruction in each MTP joint in the UD and non-UD groups to assess the relationships of bone destruction with clinical indicators and deposition of MSU crystals. The results showed that 1) there was a strong and positive correlation between the volume of MSU crystal deposition and bone erosion score; 2) bone destruction occurred in both groups, but the overall bone erosion score was significantly higher in the UD group than in the non-UD group; and 3) the UD group also had longer symptom duration and higher rate of kidney stones than the non-UD group.

In this study, the volume of MSU crystals measured from DECT images was strongly and positively associated with the degree of bone erosion, which is consistent with the results of previous studies. Jin et al. (18) evaluated MSU crystal deposits in the feet and ankles of patients at various stages of gout and found that longer disease history, presence of tophus, and bone erosion were correlated with MSU crystal deposition. In patients receiving ULT, Nicola et al. (20) demonstrated a correlation between urate depletion and skeletal erosion remodeling. These findings provide context for recent studies investigating the mechanisms of bone damage in gout. Histological studies have shown that the dysregulation of osteoclast and osteoblast functions occurs in people with erosive gout and that MSU crystals reduce osteocyte viability through direct interactions (21–23). Additionally, cells within and around the tophus produce cytokines, chemokines, enzymes, and other mediators that foster an inflammatory environment conductive to bone and joint damage (3). Innate and adaptive immune cells, including macrophages, mast cells, T and B cells, and plasma cells, have been identified within tophi (24). These studies strongly suggest that the process of gouty bone erosion is complex, with multicellular and multifactorial involvement. Moreover, the SvdH erosion scoring system, which is based on plain radiography images, is mostly used. This study found excellent inter-observer agreement for the modified SvdH erosion scoring system based on CT images, which suggests that the modified scoring system performed well on CT images.

With or without UD, bone destruction was detected in the MTP joints in this study. The UD group exhibited severer bone erosion, while small bone erosion (scored 1 or 2) was also observed in the non-UD group. This may be due to the limited detectability of DECT imaging, which may have missed tiny MSU crystals with a diameter less than 2 mm. Another possible reason for the small bone erosion is the effect of ULT. MSU crystals may have been deposited in the MTP joints before the treatment, but gradually dissolved with the course of the treatment (25, 26).

Interestingly, in contrast to a previous study by Nicola et al. (27), the serum uric acid levels were equivalent in both groups in this study. The reason for this difference may be that 100% of the patients received ULT in this study, whereas only 82% of the patients in the gout group received ULT in the aforementioned study. Therefore, there was no correlation between MSU crystals deposition, bone destruction, and serum uric levels. The relationship found in this study between serum uric acid and MSU crystal deposition is consistent with previous research (18, 28, 29). To dissolve MSU crystals and reduce clinical symptoms, guidelines worldwide consistently recommend the use of ULT to maintain serum uric acid levels ≤530 μmol/L (6, 30). However, it is difficult to assess changes in MSU deposition and bone destruction by simply measuring serum uric acid levels during clinical management because serum uric acid is a dynamic metabolite that is susceptible to the effects of drugs and diet. DECT imaging provides good reproducibility and validity (31), and is a reliable and intuitive approach for monitoring MSU deposits and observing the remodeling or expansion of bone erosion for gout management (32).

The statistically significant difference in symptom duration and history of kidney stones between the UD and non-UD groups may account for the lengthy process of tophus formation and MSU deposition in the kidneys. Tophus, a pathognomonic and hallmark feature of advanced gout that comprises MSU crystals and chronic granulomatous tissue (21, 33), typically occurs in people with gout for over 10 years (34). The most common cause of hyperuricemia is reduced renal excretion (2), which leads to MSU crystal deposits in the kidneys (35).

Although the SvdH erosion scoring system is typically used on radiography images, there was an excellent inter-observer agreement for the modified SvdH erosion scoring system based on CT images. This ensured that the modified scoring system performs well with CT images. The modified SvdH-CT erosion score was proposed in this study to detect the correlation between MSU crystal volume and erosion. This updated scoring method yielded performance consistent with that of the Rheumatoid Arthritis Magnetic Resonance Imaging Score system or Dalbeth-simplified score (29, 36) and better performance than plain radiography erosion scores (16, 36).

This study has some limitations. First, this was a retrospective study that focused only on patients with foot joint deconstruction in the MTP joints. Therefore, the results may not reflect the overall clinical situation of all gout patients, especially for those with less severe MTP joint destruction and severe joint destruction on other sites. Second, the sample size was relatively small. Third, this study was conducted in a single institution. Fourth, this scoring system performed only in MTP joints. Lastly, the study had a cross-sectional design, which means that the association between bone erosion and changes in MSU crystal volume could not be verified. Future studies comprising more joints (such as tarsometatarsal joints, ankle joints and knee joints) and a multi-center longitudinal study with a larger sample size will be conducted to validate the present findings.

To summarize, the correlation between urate volume measured from DECT images and bone erosion were evaluated. There was no correlation between MSU crystals, bone destruction, and serum uric acid. DECT can effectively monitor MSU deposits and observe changes in bone erosion, and this can be considered as a supplement to serum uric acid measurement in clinical practice to improve the prognostic evaluation of patients with gout and guide follow-up as well as individualized treatment plans.

## Data availability statement

The original contributions presented in the study are included in the article/supplementary material. Further inquiries can be directed to the corresponding author.

## Ethics statement

The studies involving human participants were reviewed and approved by Guangdong Second Provincial General Hospital. Written informed consent for participation was not required for this study in accordance with the national legislation and the institutional requirements.

## Author contributions

G-HJ: was involved in the conception and design of the study. W-YZ, W-FZ, J-YW, W-MD, Y-KH, WW: were involved in the data collection. W-YZ and J-YW: were involved in the analysis and interpretation of the data. W-YZ and W-FZ: wrote the first draft of the manuscript. J-YW and G-HJ: revising the article critically for important intellectual content. All authors contributed to the article and approved the submitted version.
